# Next Generation Ceramic Substrate Fabricated at Room Temperature

**DOI:** 10.1038/s41598-017-06774-z

**Published:** 2017-07-26

**Authors:** Yuna Kim, Cheol-Woo Ahn, Jong-Jin Choi, Jungho Ryu, Jong-Woo Kim, Woon-Ha Yoon, Dong-Soo Park, Seog-Young Yoon, Byungjin Ma, Byung-Dong Hahn

**Affiliations:** 1 Functional Ceramics Department, Korea Institute of Materials Science, 797 Changwon-daero, Seongsan-gu, Changwon, Gyeong-Nam 51508 Republic of Korea; 2 School of Materials Science and Engineering, Pusan National University, 2, Busandaehak-ro 63beon-gil, Geumjeong-gu, Busan 46241 Republic of Korea; 3 Reliability Research Center, Korea Electronics Technology Institute, 25, Saenari-ro, Bundang-gu, Seongnam-si, Gyeonggi-do 13509 Republic of Korea

## Abstract

A ceramic substrate must not only have an excellent thermal performance but also be thin, since the electronic devices have to become thin and small in the electronics industry of the next generation. In this manuscript, a thin ceramic substrate (thickness: 30–70 µm) is reported for the next generation ceramic substrate. It is fabricated by a new process [granule spray in vacuum (GSV)] which is a room temperature process. For the thin ceramic substrates, AlN GSV films are deposited on Al substrates and their electric/thermal properties are compared to those of the commercial ceramic substrates. The thermal resistance is significantly reduced by using AlN GSV films instead of AlN bulk-ceramics in thermal management systems. It is due to the removal of a thermal interface material which has low thermal conductivity. In particular, the dielectric strengths of AlN GSV films are much higher than those of AlN bulk-ceramics which are commercialized, approximately 5 times. Therefore, it can be expected that this GSV film is a next generation substrate in thermal management systems for the high power application.

## Introduction

**Figure 1:**
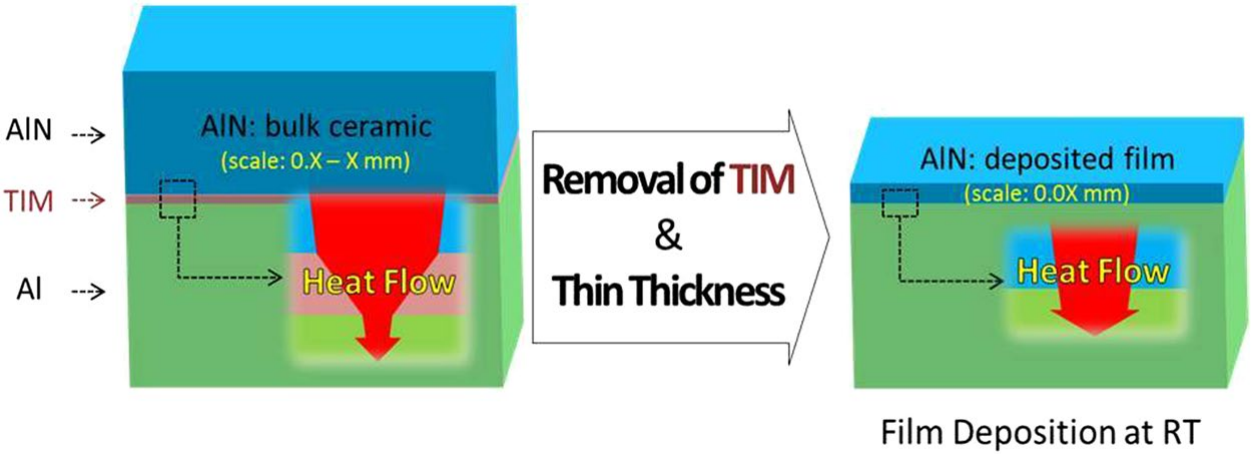
Schematic diagram of heat flow at substrate structures with TIM and without TIM. The removal of a TIM layer can improve the thermal dissipation in thermal management systems.

Ceramic materials for thermal management have gained considerable attention in the electronics industry, since the life and performance of electronic devices are dependent on the heat dissipation of electronic packages^1–8^. In general, a thermal management system consists of a ceramic substrate, a thermal interface material (TIM), and a heat sink (Al) as indicated in the left figure of Fig. 1. Among the materials, a TIM has the thermal conductivity much lower than the others^5^. In particular, a ceramic substrate must be thin, since the electronic devices have to become thin and small in the electronics industry of the next generation. Hence, we report the electric and thermal properties of AlN films which were directly coated on Al substrate without a TIM layer. AlN is the ceramic material which has high conductivity (100–270 W/mK) and it is used for a ceramic substrate in the electronics industry^6^. The AlN film had better be deposited at room temperature (RT), since the heat treatment is limited due to the easy oxidation and the low melting point (approximately 660 °C) of Al. Therefore, we designed the granule spray in vacuum (GSV) which was the room temperature process to fabricate thick films.

In this study, we report what GSV is and why GSV is required. In addition, the electric and thermal properties of AlN GSV films, deposited on Al substrates, are compared to those of commercialized structures (fabricated with bulk-AlN ceramics).

## Results

### Process Design of Granule Spray in Vacuum (GSV)

**Figure 2:**
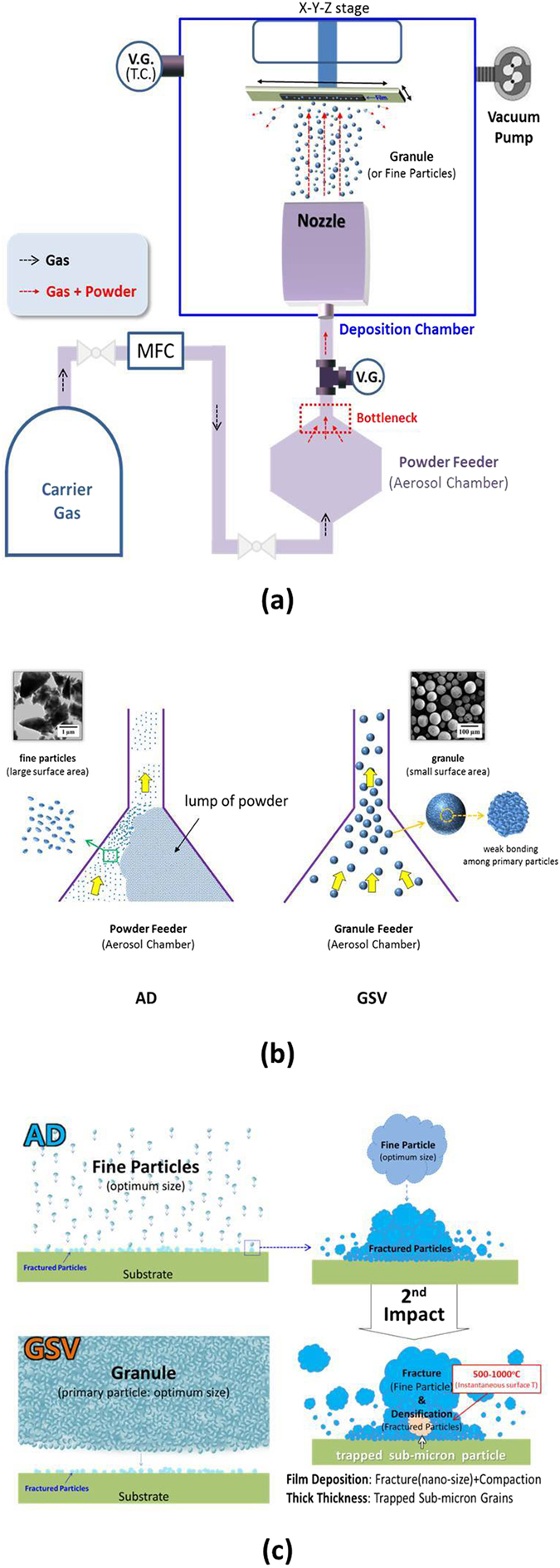
Difference between AD and GSV: (**a**) overall equipment, (**b**) bottleneck of aerosol chamber, and (**c**) deposition behavior. A lump of powders is not formed in a GSV process. For film deposition, the fine particles must be fractured to be nano-size. The compaction of particles can assist the densification of films. The film thickness is dependent on the portion of trapped sub-micron grains.

The AD method is an interesting technology which is used to fabricate a dense film at room temperature^7–17^. The fine particles, which are accelerated by an air compressor, are ejected through the nozzle and collide onto the substrate with high speed as shown in Fig. 2(a). Owing to the fracture and plastic deformation of fine particles, the dense ceramic film is formed at room temperature (RT)^14–17^. It is interesting that the dense film is formed without any heat treatment at an AD process. The AD film shows the interesting characteristics, such as the dense microstructure, nano-size grains, the high deposition speed (approximately 1–10 min for the thickness of 1 μm and the area of 10 × 10 cm^2^), the wide thickness range (from submicron to hundreds micrometers), the easy manufacture of composite films and multi-layers, the easy manufacture of flexible films (through the usage of flexible substrates), and no use of heat treatment for densification^16^. Compared to the other methods such as sputtering and pulsed laser deposition (PLD), the deposition speed of an AD method is significantly high. In addition, the grains are randomly oriented, while a PLD film shows epitaxial growth. In particular, an AD method is a room temperature process.

It has been reported that an AD film could be used for the various applications (piezoelectric devices, sensors, bio-devices, batteries, ceramic substrates etc.)^7, 8, 14–17^. However, the technology is not completed yet. In particular, the continuous flow of aerosol must be guaranteed to fabricate a uniform film using the AD method. As indicated in the left figure of Fig. 2(b), however, a lump of powder is formed at the bottleneck of a powder feeder with ease and it interferes with the aerosol flow in the AD equipment. Hence, the lump of powder must be removed for this AD method to be commercialized. The powder lump is formed due to the large surface area of fine particles. Therefore, GSV has been designed to improve the aerosol flow as seen in the right figure of Fig. 2(b). When the films are deposited by GSV, the lump of powder is not observed. At the GSV process, granules are used instead of fine particles as seen in Fig. 2(b).

In order to obtain the fracture and plastic deformation of primary particles, high kinetic energy is essential in the AD method. When the particles collide onto the substrate with high speed, they are fractured to be nano-size particles and their surface area is dramatically increased. Owing to this high surface energy and the instantaneous surface temperature of 500–1000 °C, the densification of an AD film occurs, when the other particles collide onto these nano-size particles with high speed, as shown in Fig. 2(c). It has been reported that the instantaneous surface temperature of particles and a substrate can be approximately 500–1000 °C when the particles collide onto the substrate with high speed (approximately 150–500 m/s)^14^. In general, hence, the film of a material which has a low sintering temperature is easy to be deposited in an AD or GSV process. In addition, the compaction of the fractured particles occurs due to the continuous impact of fine particles and it can assist the densification of the AD film. During this compaction process, the sub-micron-size particles are frequently trapped in AD films, as shown in Fig. 2(c).

**Figure 3:**
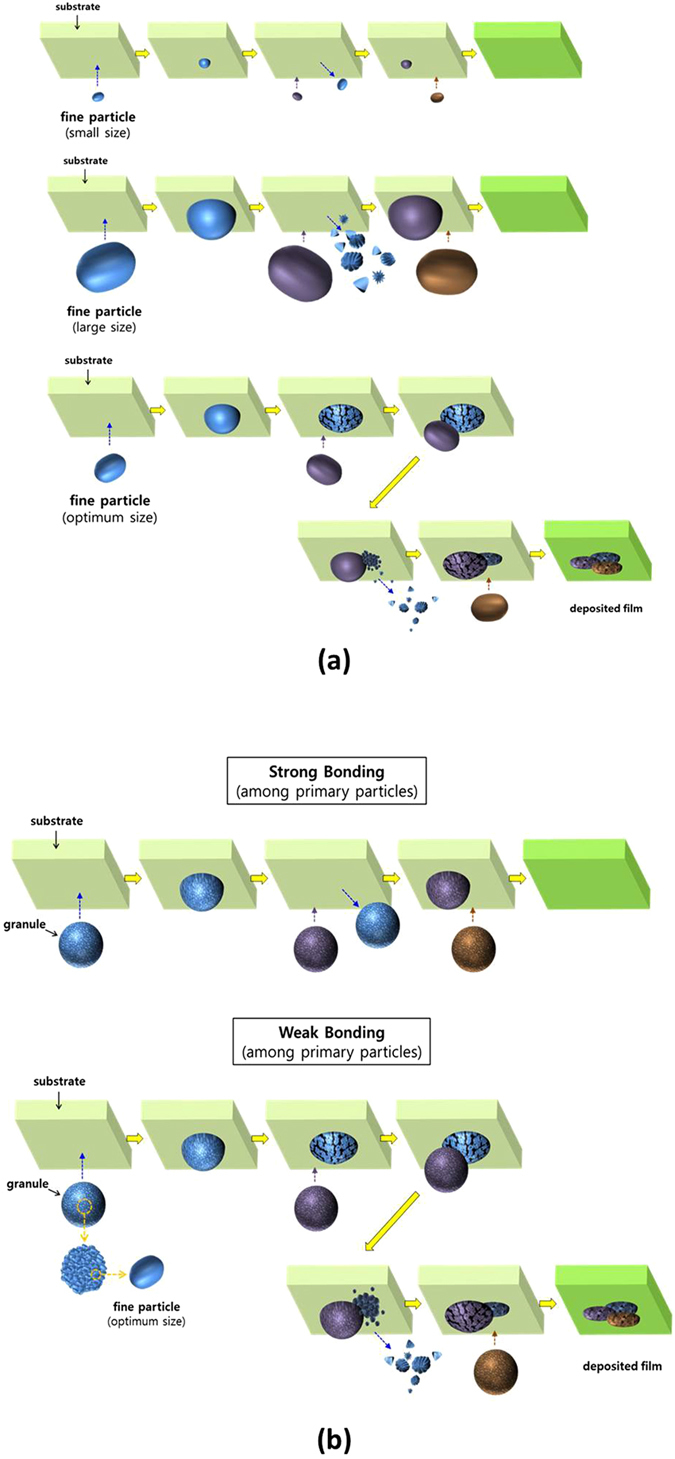
Deposition behaviors in (**a**) AD and (**b**) GSV. The AD films can be formed by using the optimum particle size. The GSV films can be formed by using the fragile granules.

**Figure 4:**
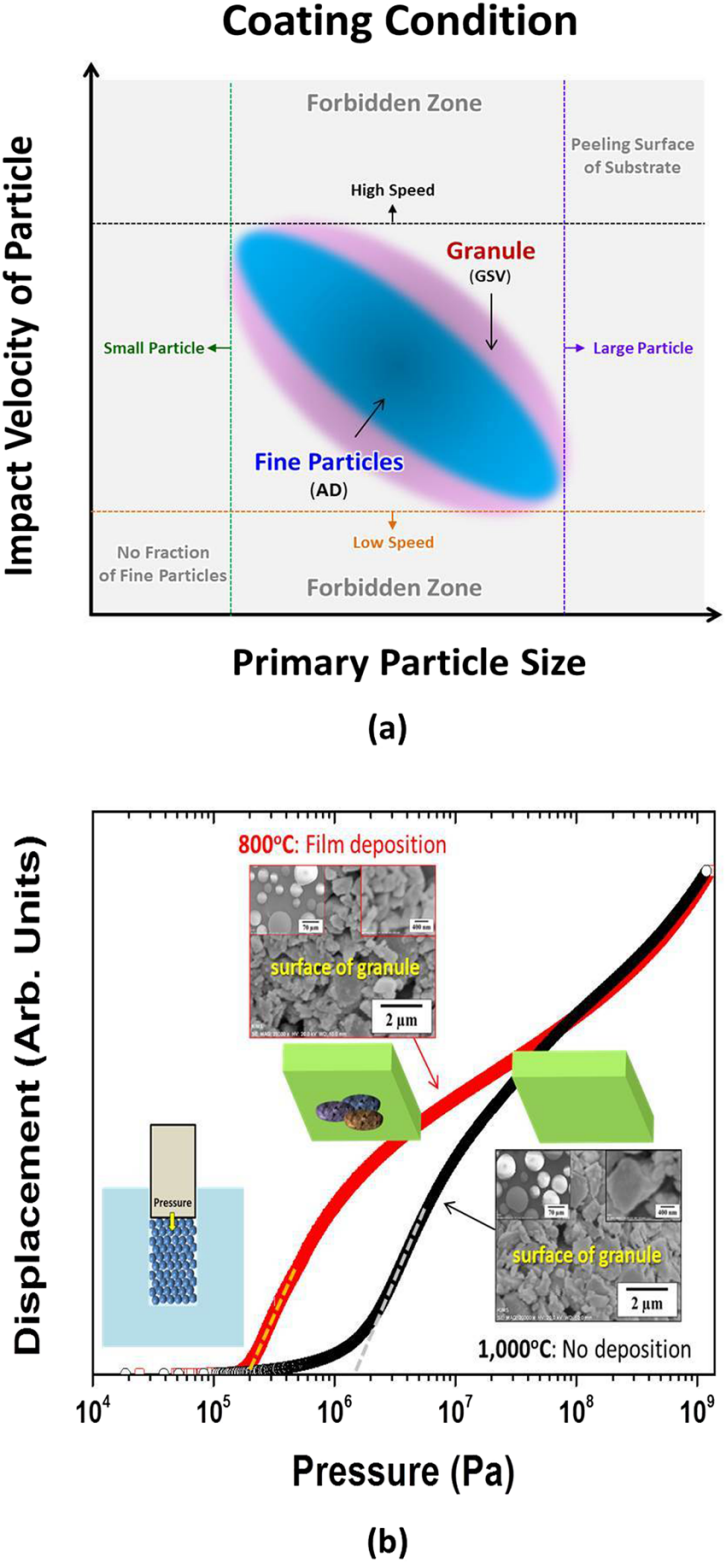
Coating conditions for AD and GSV. In a GSV process, the coating condition is wider than that in an AD method, owing to the wide size distribution of fractured particles. The granules are fragile when they were heat-treated at 800 °C, compared to those heat-treated at 1000 °C. Their SEM images are not significantly different.

The fine particles must be fractured by their kinetic energy for AD films to be deposited on the substrates, as explained above. If the kinetic energy is not enough for the particles to be fractured (due to the light mass or the low speed of particles), the particles will not be fractured as shown in the top figure of Fig. 3(a). The light mass means the small particle in Fig. 4(a). On the contrary, the surface of a substrate can be peeled by the high impact energy of large particles, when the kinetic energy is immoderate (owing to the heavy mass or the high speed of particles). Even though the particles are fractured, the size of fractured particles is too large to be deposited on the substrate, as indicated in the middle figure of Fig. 3(a). In order to be well-deposited on the substrate, the fractured particles must be nano-sized as explained above. Although the small amount of nano-size particles can be existed on a substrate, they are easily removed by the continuous impact of large fine-particles. Therefore, the optimum size and velocity of fine particles must be used for the AD films to be successfully deposited, as marked with the blue color in Fig. 4(a). A gray area is the forbidden zone in which the film is not deposited. The optimum condition is dependent on the kind of materials. When the conditions are satisfied, the AD films are deposited as shown in the bottom figure of Fig. 4(a).

This AD process must be significantly improved as shown in Fig. 2(b), although the AD films can be deposited by considering those conditions. The continuous flow of aerosol can be guaranteed by using granules instead of fine particles, as mentioned above. The granules consist of the fine particles which have the optimum size as exhibited in the bottom figure of Fig. 3(b). In the GSV process, the deposition of GSV films is dependent on the fracture of primary particles. The surface of a substrate can be peeled due to the high impact energy of large granules, if the primary particles cannot be fractured by the impact of granule as shown in the top figure of Fig. 3(b). This hard granule is formed by the strong bonding among primary particles. Therefore, the primary particles must be weakly bonded to be easily fractured, in the fragile granules, for the GSV films to be deposited on a substrate as shown in the bottom figure of Fig. 3(b). The bonding strength (among primary particles) can be controlled by the annealing temperature of granules as seen in Fig. 4(b). The higher temperature means the harder granule. The difference of the bonding strength was measured as indicated in the inset of Fig. 4(b). As shown in Fig. 4(b), the granules did not show the significant difference at the SEM images, but their bonding strengths were severely different. The granules, hence, which were heat-treated at 800 °C, might be well-fractured at a GSV process, while they could not be well-fractured when they were heat-treated at 1000 °C. Owing to this tendency of fracture, the AlN GSV films could be well-formed on the Al substrate, when the granules were heat-treated at 800 °C. In general, the optimum temperature is also dependent on the kind of materials and the size of primary particles.

In general, as seen in Fig. 4(a), the coating condition is wider in a GSV process than in an AD process, although the difference is not significant. The difference is due to the size distribution of fractured particles. The granules have the kinetic energy much higher than fine particles, owing to their heavy masses. This high kinetic energy is principally consumed by the fracture of a fragile granule. In addition, the fracture of primary particles occurs from the particles which are close by the impact surface. Therefore, the size distribution of fractured particles is wider in a GSV process than in an AD process. This wide size distribution of fractured particles makes the coating condition wider as shown in Fig. 4(a). Furthermore, the trapped sub-micron particles [Fig. 2(c)] are also observed in GSV films more than in AD films. Owing to the huge size of granules [compared to fine particles, as shown in Fig. 2(c)] and the wide size distribution of fractured particles, the sub-micron particles can have chance to be trapped among the nano-size particles in a GSV process more than in an AD process. For the compaction of the fractured particles, granules are also more effective than fine particles.

### Crystal Structure and Microstructure of AlN GSV Film

**Figure 5:**
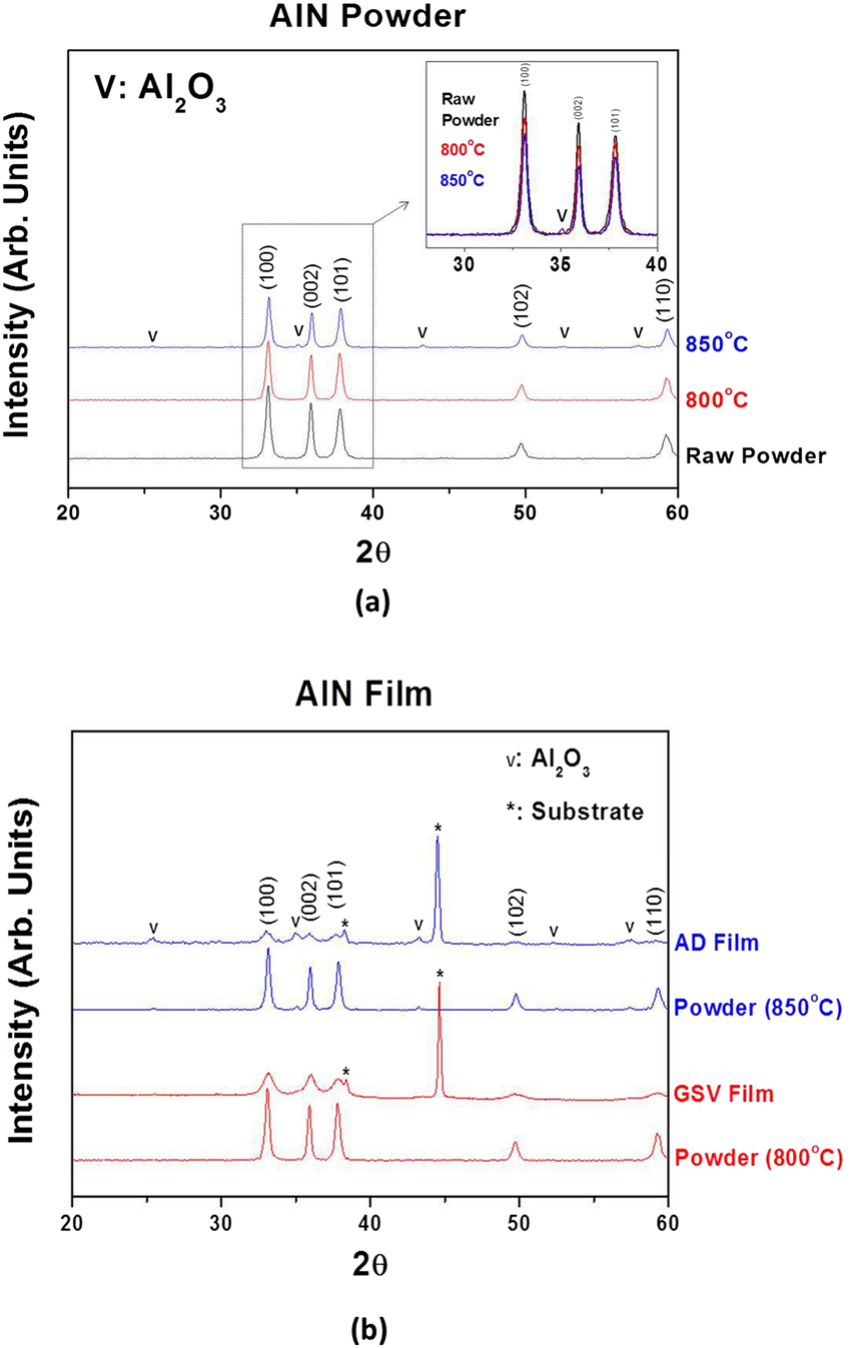
XRD patterns of (**a**) AlN powders and (**b**) AlN AD/GSV films. The peaks for Al_2_O_3_ are observed when they are heat-treated at 850 °C.

Figure 5 shows the X-ray diffraction (XRD) patterns of AlN powders (heat-treated at 800 °C and 850 °C in air) and AlN films (AD and GSV, deposited on Al substrates). As seen in Fig. 5(a), the Al_2_O_3_ peaks were not detected at the XRD patterns, when AlN powers were heat-treated at 800 °C (AlN 800). On the contrary, they were found at the XRD patterns of the AlN powders which were heat-treated at 850 °C (AlN 850). The peak intensity for AlN decreased with the rise of heat-treatment temperature as seen in the inset of Fig. 5(a). Moreover, the peaks of Al_2_O_3_ were detected at AlN 850. Hence, the surface of AlN 800 might be also oxidized, although the oxidation rate was negligible.

**Figure 6:**
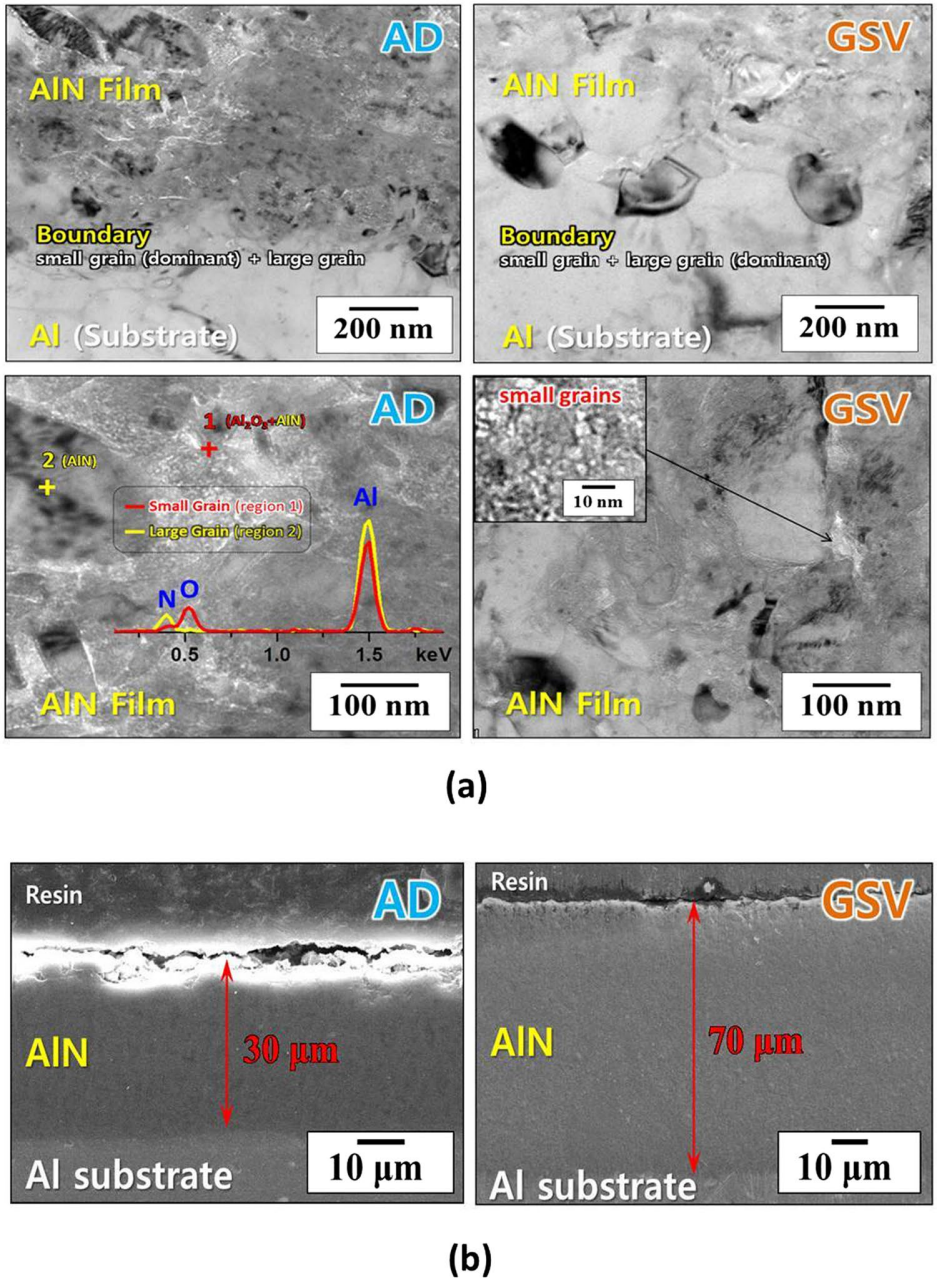
(**a**) TEM and (**b**) SEM images of AlN AD film and AlN GSV film. The small Al_2_O_3_ grains are observed among the large AlN grains in AlN AD films more than in AlN GSV films. primary particles. The GSV films can be formed by using the fragile granules. The AlN films show the dense microstructures. It is difficult for AlN AD films to be deposited thicker than 30 µm.

In an AD process, AlN 850 had to be used for the AlN AD films to be well-deposited on an Al substrate. Using AlN 800, it was difficult to obtain the thick AlN AD film which was thicker than 10 µm. In addition, we have tried to deposit the AlN AD films using pure AlN powders after controlling their particle sizes and phases (no Al_2_O_3_, heat-treated in N_2_ and H_2_ atmosphere), and then the film thickness was limited to be <5 µm. Therefore, pure AlN was the material which was difficult to be deposited with thick thickness by an AD process. On the contrary, it is well-known that Al_2_O_3_ is well-deposited by an AD method^13, 14^. Namely, the Al_2_O_3_ (formed at the surface of AlN 850 particles) must be required for AlN AD films to be well-formed on an Al substrate (thicker than 5 µm). Consequently, the AD film of an AlN-Al_2_O_3_ composite was deposited by the AD process as indicated in the blue line of Fig. 5(b) and the left figures of Fig. 6(a). As mentioned above, the formation of AD or GSV films is easy when the sintering temperature of the coating material is low. In bulk ceramics, the general sintering temperature of Al_2_O_3_ is 1,500–1,600 °C, while AlN must be sintered at 1,900–2,000 °C. Therefore, it is a natural phenomenon that Al_2_O_3_ is deposited with ease, compared to AlN.

The microstructure of this composite film was indicated in the left figures of Fig. 6(a). The nano-size grains of Al_2_O_3_ and AlN were formed at the grain boundary among the sub-micron grains of AlN. This AlN AD film showed the large portion of trapped sub-micron grains [Fig. 2(c)], compared to the AD films of the other materials which have been reported until now^14–17^. In the microstructure of normal AD films, the sub-micron grains are rarely observed. Moreover, in general, it is difficult to obtain the AD films which is thicker than tens-of-micrometer due to the compressive stress which occurs owing to the nano-size grains^17^. Therefore, the portion of trapped submicron-size grains must be increased in order to obtain thick AD films and this AlN AD film shows the large portion of trapped sub-micron grains as seen in the left figures of Fig. 6(a).

The dense microstructures of AlN AD films were observed as seen in the left figure of Fig. 6(b). However, it was difficult for an AlN AD film to be deposited thicker than 30 µm. The deposition of thick AlN AD films is due to the formation of trapped sub-micron particles, as mentioned above. Hence, in order to make the thickness of AlN films thicker than 30 µm, the portion of trapped sub-micron particles must be larger than that in AlN AD films. As explained above, the sub-micron particles can have chance to be trapped among the nano-size particles in a GSV process more than in an AD process. Therefore, the AlN GSV films was deposited on an Al substrate and their microstructures were compared to those of the AlN AD films as shown in Fig. 6. As seen in these figures, in AlN GSV films, the portion of sub-micron grains was larger than that in AlN AD films and the AlN GSV films were well-deposited with the thickness of 70 µm. Therefore, the difference of film thickness (between an AlN GSV film and an AlN AD film) is due to the different portion of sub-micron grains. It was interesting that AlN GSV films were well-deposited by AlN 800 in which the oxidation of AlN was negligible as shown in the red lines of Fig. 5(b) and the right figures of Fig. 6(a). Since the amount of Al_2_O_3_ (formed on the surface of AlN 800) was much smaller than that of AlN 850, the region of nano-size grains became thinner as seen in Fig. 6, when the GSV process was used to deposit AlN films. In addition, the AlN GSV films can be deposited on an Al substrate by using AlN 800, since the coating condition is wide in a GSV process, compared to that in an AD process, as indicated in Fig. 4(a).

The anchoring layers, which permit the strong adhesion between Al substrates and AlN films, are observed in both films, as seen in the top figures of Fig. 6(a) ^14^. 5052 Al contains the elements of Mg (2.2–2.8 wt.%) and Cr (0.15–0.35 wt.%). Hence, it could be also considered that Mg and Cr, which were located on the surface of an Al substrate, might lead to preferential interactions with the deposition layer. In addition, these could lead to the formation of nitrides which might provide additional adhesion. The formation of nitrides and the additional adhesion were not identified in this study. Furthermore, although the films were immediately deposited after polishing, a thin oxide layer could be formed. However, the thickness was too thin to be observed in TEM images.

### Electric and Thermal Properties of AlN GSV Film

**Figure 7:**
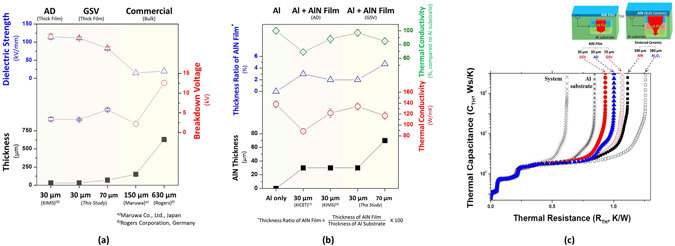
(**a**) Electric and (**b**,**c**) thermal properties of AlN AD films and AlN GSV films. The thicker the film thickness is, the higher the breakdown voltage is. The dielectric strength of an AlN GSV film is much higher than that of an AlN bulk-ceramic which is commercialized. The thermal properties of AlN GSV films are better than those of the others.

Figure 7(a) shows the electric properties of AlN AD films and AlN GSV films. As seen in this figure, although their thicknesses were thin compared to those of the AlN bulk-ceramics (a few hundred micro-meters), the breakdown voltages were very high enough. In particular, the dielectric strengths of AlN films were much higher than those of AlN bulk-ceramics which have been commercialized in industry, as shown in Fig. 7(a). It might be due to the dense microstructure and the space charge in the grain boundary. The AlN AD (or GSV) films showed the nano-size grains among the sub-micron grains as seen in Fig. 6(a). In addition, AlN AD films exhibited the break-down voltage higher than that of AlN GSV films, owing to the large portion of nano-size grains in AlN AD films, as seen in Figs 6(a) and 7(a). Likewise, the thicker the films are, the higher the break-down voltages become, as shown in Fig. 7(a). On the contrary, the dielectric strength decreased with increasing the thickness of AlN GSV films as indicated in Fig. 7(a). This thickness dependence on the dielectric strength has been reported to be commonly found in dielectric materials^8, 18^.

The thermal properties of AlN AD films and AlN GSV films were exhibited in Fig. 7(b). In general, for the thin films (less than several micrometers in thickness), the 3ω method is used to measure their thermal conductivities^19^. However, there is no appropriate method to measure the thermal conductivities of thick ceramic films (the thickness range between 10 µm and 100 µm). Hence, the thermal properties of AlN-film-coated Al substrates (AD Al and GSV Al) were measured by the laser flash method. AD Al and GSV Al showed the thermal conductivities (117–134 W/mK, 85–97% compare to that of an Al substrate) lower than that of an Al substrate (138 W/mK), although it was well-known that the thermal conductivity of AlN was high (approximately 100–270 W/mK in bulk-ceramics)^6, 20^. It means that AlN AD films and AlN GSV films can show the thermal conductivities lower than Al substrate. It has been reported that the low conductivities of the AlN films were due to their small grains^9–12^. The grain size of AlN bulk-ceramics is larger than several micrometers^7^. In addition, the thermal conductivity of Al_2_O_3_ bulk-ceramics is low approximately 20–30 W/mK, while AlN bulk-ceramics have the high thermal conductivity higher than 100 W/mK^6, 21^. Hence, the formation of Al_2_O_3_ grains might be also responsible for the low thermal conductivities of AD Al and GSV Al as seen in Figs 6(a) and 7(b). Owing to the low portion of Al_2_O_3_ grains in AlN GSV films, therefore, the thermal conductivity of GSV Al (134 W/mK) was higher than that of AD Al (122 W/mK), when the thickness was 30 µm, as seen in the left figure of Fig. 7(b). Increasing the thickness from 30 µm to 70 µm, the thermal conductivity of GSV Al decreased from 134 W/mK to 117 W/mK. This decrease of thermal conductivity is due to the thermal conductivities of AlN GSV films lower than those of Al substrates. The thermal conductivities of AlN GSV films can be further improved by laser annealing as seen in Figs. S1–S4.

Their thermal conductivities were compared to those of AlN AD films which have been reported until now, as indicated in Fig. 7(b) ^7^. Heo *et al*. have reported that the excellent thermal property was observed at the structure which consisted of an AlN AD film and an Al substrate without TIM, compared to the commercialized substrates. The AD Al and GSV Al exhibited the thermal conductivities much higher than that of the previous report, although Heo *et al*. used an AlN powder which had high quality (H-grade, Tokuyama). Considering their commercialization, in this study, the AlN AD films and the AlN GSV films were produced by the AlN powder which had low cost (Eno Material, Qinhuangdao, China; oxygen content: 0.8 wt.%), compared to the H-grade AlN powder of Tokuyama. The difference between Heo’s film and our films is the Al_2_O_3_ layer between an AlN film and an Al substrate. Heo *et al*. have formed the Al_2_O_3_ layer (thickness: 1 µm) between an AlN film and an Al substrate, in order to form the AlN films with ease and to prevent an Al substrate from oxidation during the heat treatment of 500 °C. They have explained that the heat treatment of 500 °C was required to form the silver electrode on the AlN film. However, AlN AD films and AlN GSV films were well-deposited on Al substrates without the Al_2_O_3_ layer at room temperature, in this study. In addition, the AD Al and GSV Al were heat-treated at 500 °C as a trial, but any difference was not found in electric and thermal properties before and after heat-treatment. Most of all, the Al_2_O_3_ film between an Al substrate and an AlN film must make the significant decrease of thermal properties, since it is similar to the TIM layer of a bulk-based commercial structure. On the contrary, although the thin oxide layer can be also formed at the boundary between an Al substrate and an AlN film in this study, it is too thin to be observed in TEM images. Therefore, the thermal properties were not decreased significantly, compared to those of Heo’s specimens, as seen in Fig. 7(b).

**Figure 8:**
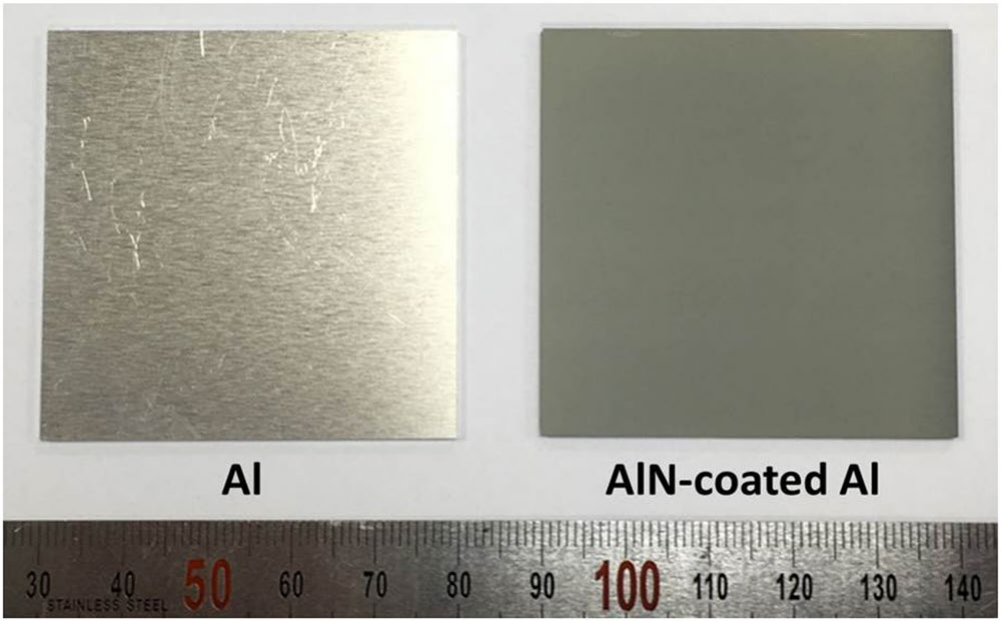
Photographs of Al substrate and AlN GSV film.

Figure 7(c) shows the thermal resistances of various structures. As seen in this figure, the structure of AD Al and GSV Al exhibited the thermal resistance lower than the commercialized structure which consisted of a bulk-AlN ceramic, a TIM, and an Al substrate (product of Amosense, Korea). In particular, the thermal resistance of a GSV Al was lower than that of an AD Al [the 30 µm specimens in Fig. 7(c)]. This difference of thermal resistances is due to their microstructures as explained above. In AlN GSV films, the large AlN grains were observed much more than in the AlN AD films. The photograph of a GSV Al (5 cm × 5 cm) was exhibited in Fig. 8. Using our equipment, the size of 1 m × 1 m can be produced now.

## Summary

A TIM hinders the thermal dissipation at the thermal management system which consists of a ceramic substrate, TIM, and a heat sink (Al). In addition, the thin ceramic substrate is required, since the electronic devices have to become thin and small in the electronics industry of the next generation. Hence, we have designed the structure which consisted of a ceramic film and an Al substrate without a TIM layer. For the ceramic material, AlN was chosen, since it was well-known that AlN showed high thermal conductivity, compared to the other ceramic materials. The AlN films were fabricated at RT using an AD method and GSV, owing to the low melting point of Al. The deposition mechanism of GSV is not different from that of AD method. In the GSV process, granules are used instead of fine particles which are used in the AD process. Owing to the type of particles, the continuous flow of aerosol is guaranteed in a GSV process, while a lump of powder is formed at the bottleneck of a powder feeder with ease and it interferes with the aerosol flow in the AD equipment. The film deposition also became easier in a GSV process. AlN GSV films were deposited on Al substrates and their electric and thermal properties compared to those of AlN AD films. The thermal properties of GSV films were better than those of AD films. In particular, AlN GSV films showed the dielectric strengths much higher than those of AlN bulk-ceramics which were commercialized, approximately 5 times. Moreover, the thermal resistance was significantly improved by the removal of a TIM in thermal management systems. Therefore, it could be expected that the structure, which consisted of an AlN GSV film and an Al substrate, might be a promising candidate in thermal management systems for the high power application.

## Methods

A AlN powder (Eno Material, Qinhuangdao, China; oxygen content: 0.8 wt.%) was used for an AD process. The powder is a cheap powder which is commercially available. The AlN powder was milled using a planetary ball-miller for 4 h. For AlN AD films, the slurry was then dried and heat-treated at 800–850 °C for 2 h in air. For AlN GSV films, the powder was mixed with a polyvinyl butyral (PVB). Using this slurry, AlN granules were prepared by a spray dryer. The granules were heat-treated at 800–1000 °C for 2 h. AlN thick-films were deposited on an Al substrate (5052 Al plates, diameter: 12.7 mm, thickness: 1.5 mm) by an AD method and a GSV process as indicated in Fig. 2. The AlN films were immediately deposited after the surface polishing of Al (surface roughness: <1 µm). The air, as a carrier gas, was flowed into the aerosol chamber at the flow rate of 30 L/min. A slit-type nozzle (35 mm × 1 mm^2^) was used.

The crystal structure was determined using an X-ray diffractometer (XRD, D-MAX 2200, Rigaku Co., Tokyo, Japan). Scanning electron microscopy (SEM, JSM-5800, JEOL Co., Tokyo, Japan) and transmission electron microscope (TEM, JEM 2100 F; JEOL CO., Tokyo, Japan) were used to observe the microstructures of the films. The electric breakdown voltages of the films were measured using a withstanding voltage tester (TOS 5101, Kikusui Electronics Corp., Yokohama, Japan). The voltage was applied at the rate of 500 V/s and five samples were tested. The thermal diffusivity of the AlN-coated Al substrate was determined at room temperature by the laser flash method using a laser flash unit (LFA 457, MicroFlash, Netzsch Instruments Inc., Germany). The thermal conductivities of the AlN-coated Al samples were calculated from the thermal diffusivity, specific heat, and density of the specimens. Thermal transient measurement of the samples was also carried out with the thermal transient tester (T3Ster, Mentor Graphics Inc., OR, USA) in connection with the DynTIM tester (Mentor Graphics Inc., OR, USA).

## Electronic supplementary material

Next Generation Ceramic Substrate Fabricated at Room Temperature
