# Supplementary material for: Next Generation Ceramic Substrate Fabricated at Room Temperature
Source: Sci Rep. 2017 Jul 26;7:6637. doi: 10.1038/s41598-017-06774-z (PMC5529459; doi:10.1038/s41598-017-06774-z)
Supplement: Supplementary file 1 — Next Generation Ceramic Substrate Fabricated at Room Temperature [file 41598_2017_6774_MOESM1_ESM.pdf]

Supplementary Information

Next Generation Ceramic Substrate Fabricated at Room Temperature

Yuna Kim[1,2], Cheol-Woo Ahn[1], Jong-Jin Choi[1], Jungho Ryu[1], Jong-Woo Kim[1], Woon-Ha Yoon[1], Dong-Soo Park[1], Seog-Young Yoon[2], Byungjin Ma[3], Byung-Dong Hahn[1**]*

[1]*Functional Ceramics Department, Korea Institute of Materials Science, 797 Changwon-daero, Seongsan-gu, Changwon, Gyeong-Nam, 51508, Republic of Korea*
[2]*School of Materials Science and Engineering, Pusan National University, 2, Busandaehak-ro 63beon-gil, Geumjeong-gu, Busan, 46241, Republic of Korea*
[3]*Reliability Research Center, Korea Electronics Technology Institute, 25, Saenari-ro, Bundang-gu, Seongnam-si, Gyeonggi-do, 13509, Republic of Korea*

E-mail: cheoruahn@kims.re.kr, cera72@kims.re.kr

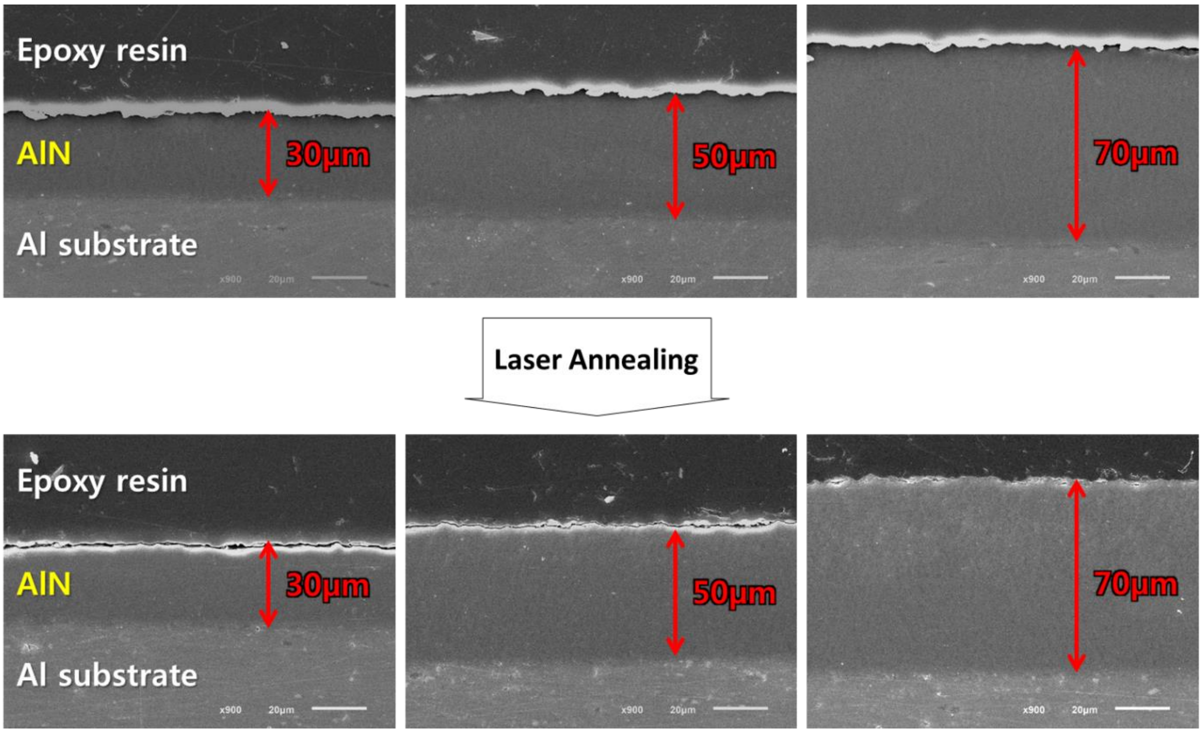

Figure S1. Variation of SEM images by laser annealing in AlN GSV films. All of the films showed dense microstructures. Any variation was not found in the SEM images of AlN GSV films after laser annealing.

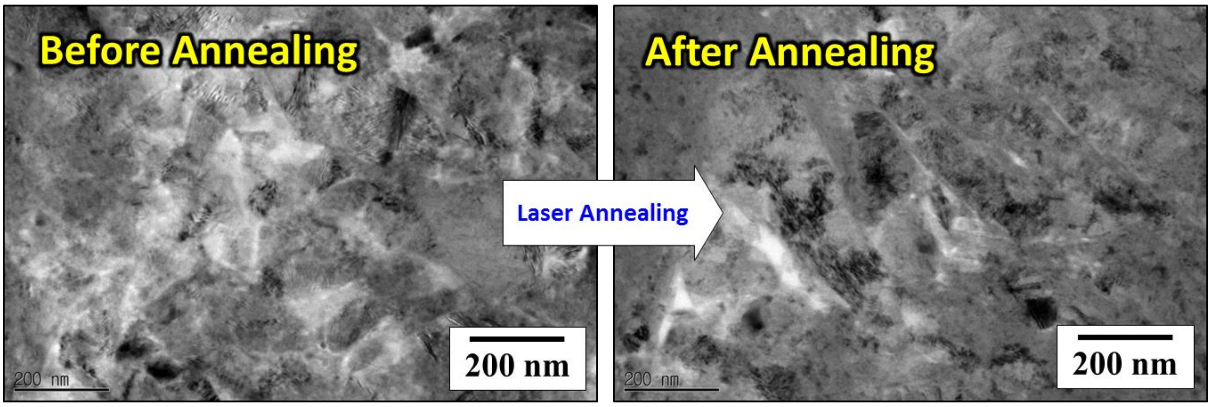

Figure S2. Variation of TEM images by laser annealing in AlN GSV films. The difference of grain size was not significant in TEM images.

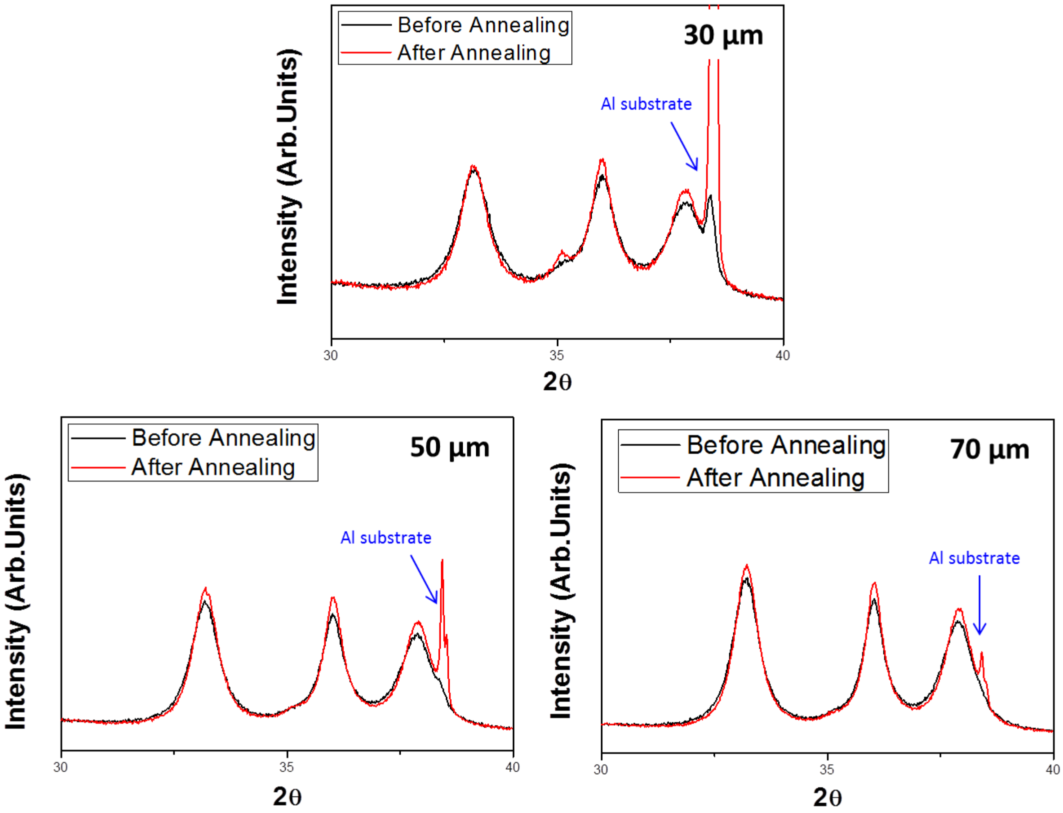

Figure S3. Variation of XRD patterns by laser annealing in AlN GSV films. The large AlN grains might be grown by the dissolution of the small AlN grains which had been existed among the large AlN grains before laser annealing. The grain growth occurred, but it was not significant. The effect of laser annealing on an Al substrate was dependent on the thickness of AlN GSV films. The laser annealing could make an Al substrate recrystallized.

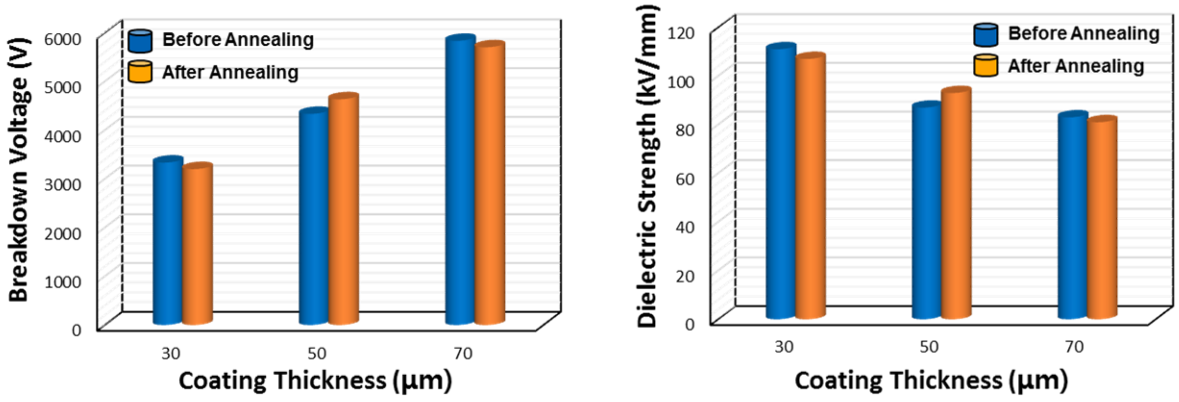

(a)

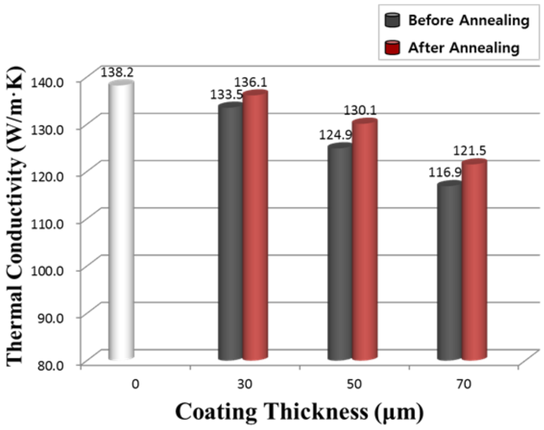

(b)

Figure S4. Variation of (a) electric and (b) thermal properties by laser annealing in AlN GSV films. The significant variation of electric properties was not observed in AlN GSV films after laser annealing. The thermal properties of laser-annealed specimens were better than those of the specimens which were not laser-annealed, since the laser annealing made the grain size grown.
